# Supplementary material for: β-III tubulin identifies anti-fibrotic state of pericytes in pulmonary fibrosis
Source: Res Sq. 2025 Dec 9:rs.3.rs-8138421. Preprint. [Version 1] doi: 10.21203/rs.3.rs-8138421/v1 (PMC12747291; doi:10.21203/rs.3.rs-8138421/v1)
Supplement: 1 [file NIHPPrs8138421v1-supplement-1.pdf]

Figure S1.

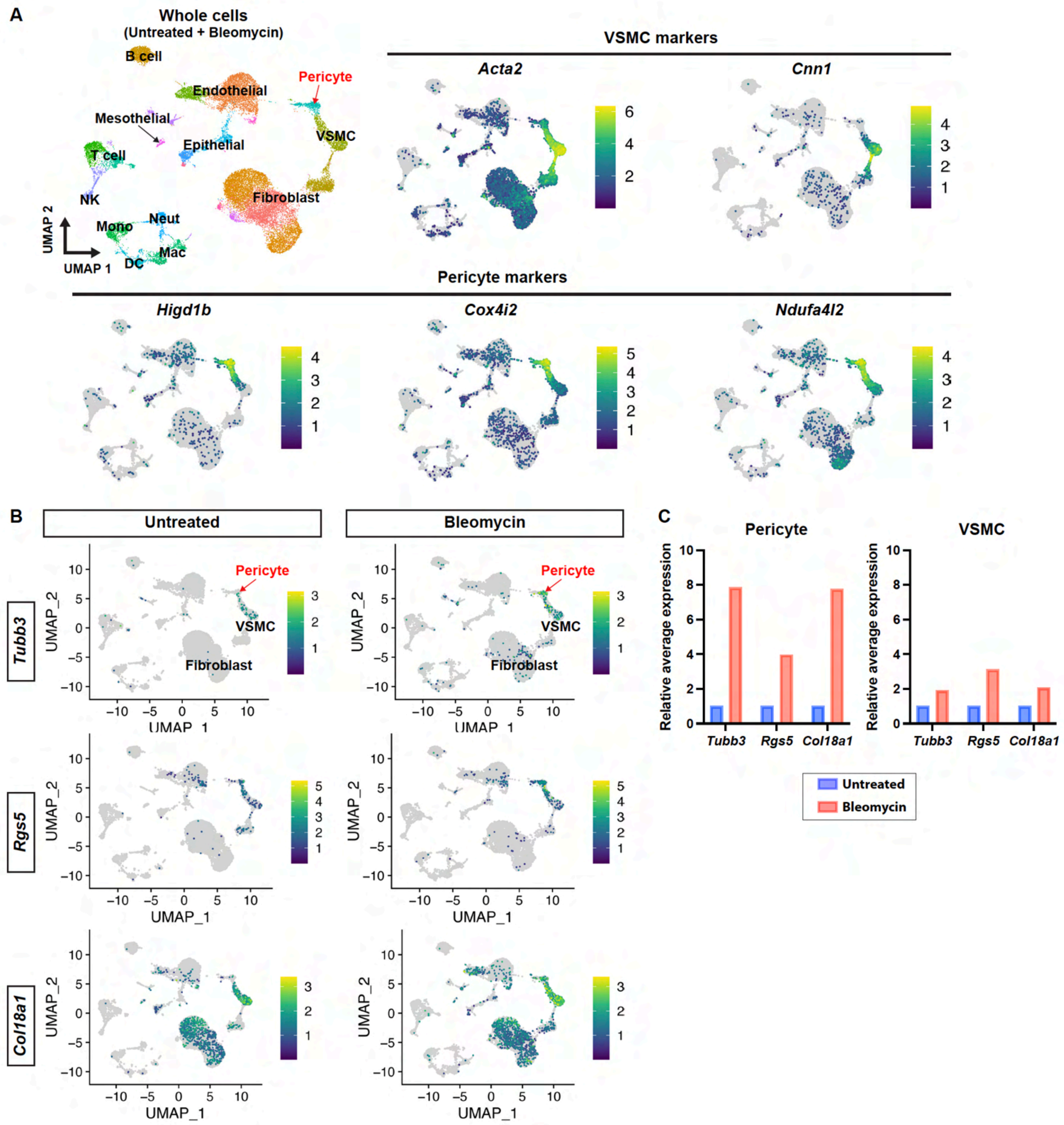

Figure S1. **Bleomycin-induced lung injury selectively upregulates *Tubb3* expression in lung pericytes.**

(A) UMAP plot of all cells from both untreated and bleomycin-treated samples, with cell type annotations, based on the scRNA-seq dataset from *Tsukui et al., 2020*[25]. Expression of VSMC marker genes (*Acta2* and *Cnn1*) and pericyte marker genes (*Higd1b*, *Cox4i2*, and *Ndufa4l2*) is shown in UMAP plots. **(B)** UMAP plots split by treatment conditions (untreated and bleomycin-treated) showing expression of activated pericyte marker genes (*Tubb3*, *Rgs5*, and *Col18a1*) across all cells. **(C)** Comparison of the

average expression of activated pericyte marker genes in pericyte (left) and VSMC (right) populations between untreated (blue) and bleomycin-treated (red) samples. Y-axis shows fold change in average expression relative to untreated samples (set to 1). VSMC: vascular smooth muscle cell; NK: natural killer cell; Mono: monocyte; Neut: neutrophil; Mac: macrophage; DC: dendritic cell.

Figure S2.

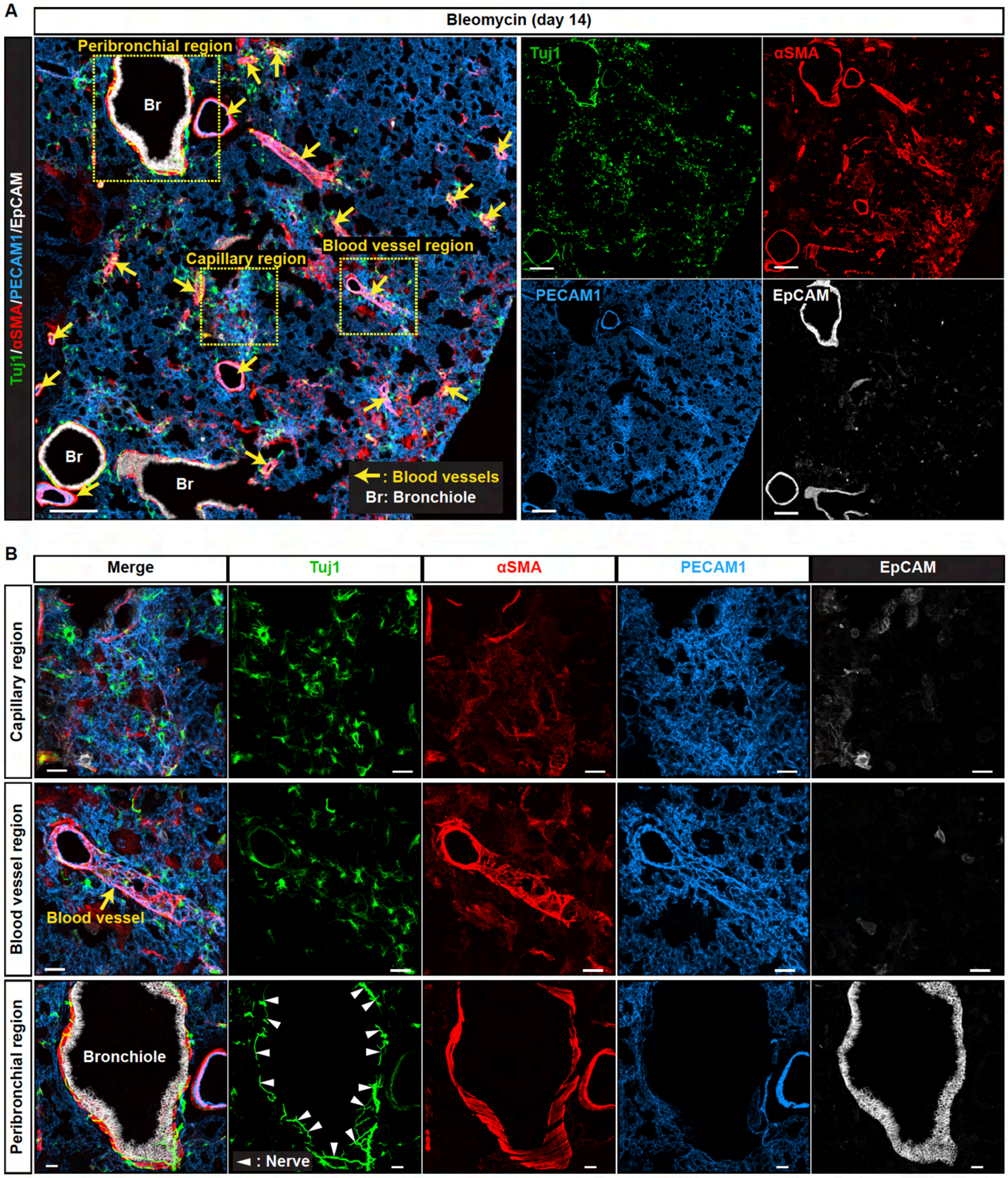

Figure S2. **Tuj1 expression patterns in vascular and bronchiolar regions of the bleomycin model. (A)** Immunostaining of bleomycin-induced fibrotic lungs (day 14) with Tuj1 (green), αSMA (red), PECAM1 (blue), and EpCAM (gray). Boxed regions are magnified in the panels in (B) to highlight

capillary, blood vessel, and peribronchial regions. Scale bar, 200 μm. **(B)** Magnified images of capillary region ($PECAM1^+$, $EpCAM^-$, without αSMA^+ vascular smooth muscle cell coverage; upper panels), blood vessel region ($PECAM1^+$, $EpCAM^-$, with αSMA^+ vascular smooth muscle cell coverage; middle panels), and peribronchial region ($PECAM1^-$, $EpCAM^+$, with αSMA^+ airway smooth muscle cell coverage; lower panels). Arrow heads show the nerves surrounding the bronchiole. Scale bars, 30 μm.

Figure S3.

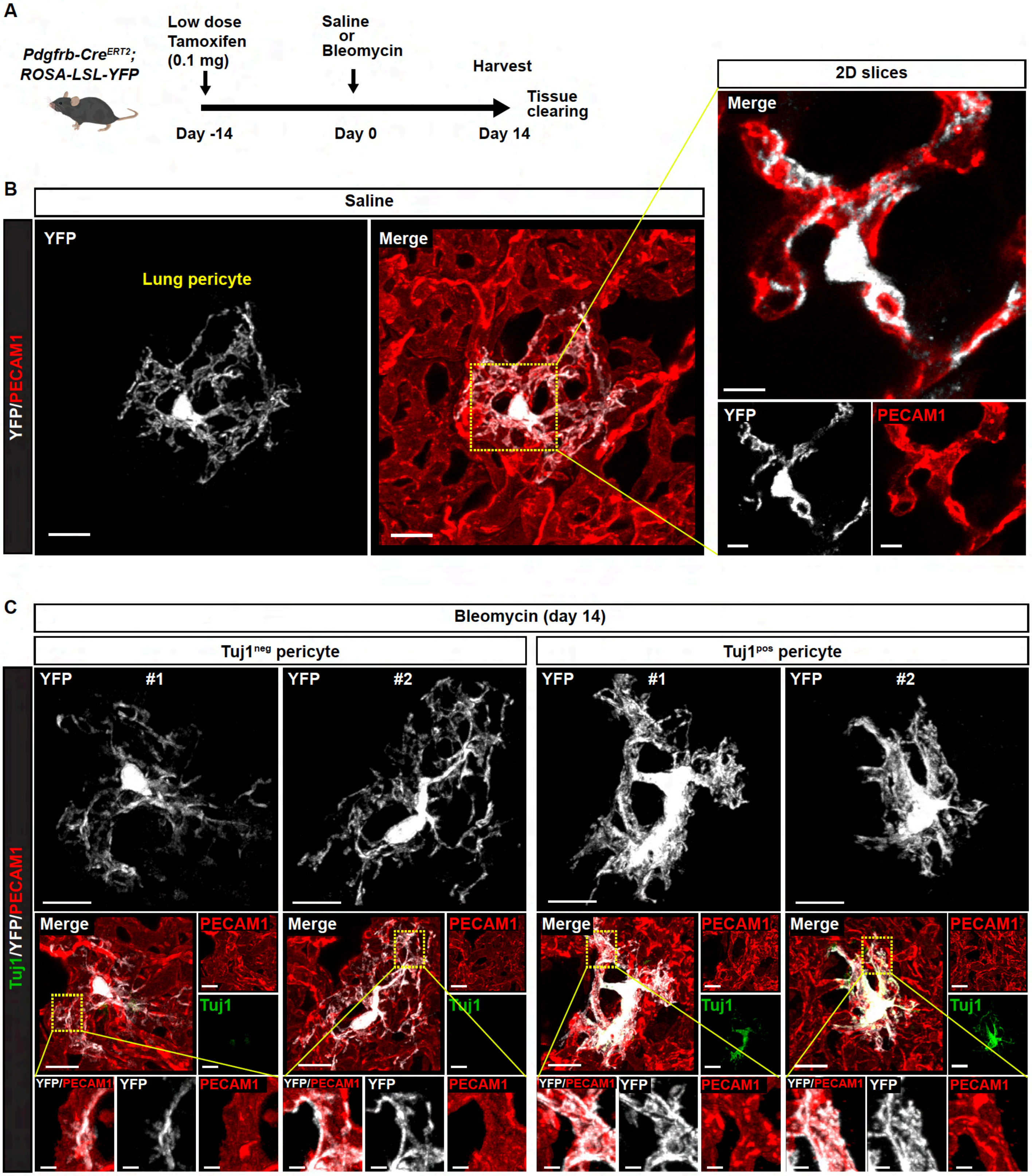

Figure S3. **Cellular morphology of single lung pericytes in homeostatic and bleomycin-induced fibrotic conditions.**

(A) Schematic illustration of mosaic pericyte labeling using *Pdgfrb-Cre^{ERT2}; ROSA-LSL-YFP* mice. A single low-dose tamoxifen (0.1 mg) was administered before saline or bleomycin treatment, with lung

tissues harvested on day 14. **(B)** Immunostaining of tissue-cleared lungs from saline-treated *Pdgfrb-Cre^{ERT2}; ROSA-LSL-YFP* mice with YFP (gray) and PECAM1 (red). Attenuated-maximum intensity projection (attenuated-MIP) was used to visualize 3D lung vascular structures. Right panels: magnified 2D slice images of boxed regions in the left panel. Scale bars, 10 μm (left panels); 5 μm (right panels). **(C)** Immunostaining of tissue-cleared lungs from bleomycin-treated *Pdgfrb-Cre^{ERT2}; ROSA-LSL-YFP* mice with YFP (gray), Tuj1 (green), and PECAM1 (red). Representative images of Tuj1^{neg} and Tuj1^{pos} pericytes are shown (two cells per phenotype). Boxed regions in attenuated-MIP rendered images of Tuj1^{neg} and Tuj1^{pos} pericytes (middle panels) are magnified as corresponding 3D cropped images on the bottom panels. Scale bars, 10 μm; 2 μm (3D cropped).

Figure S4.

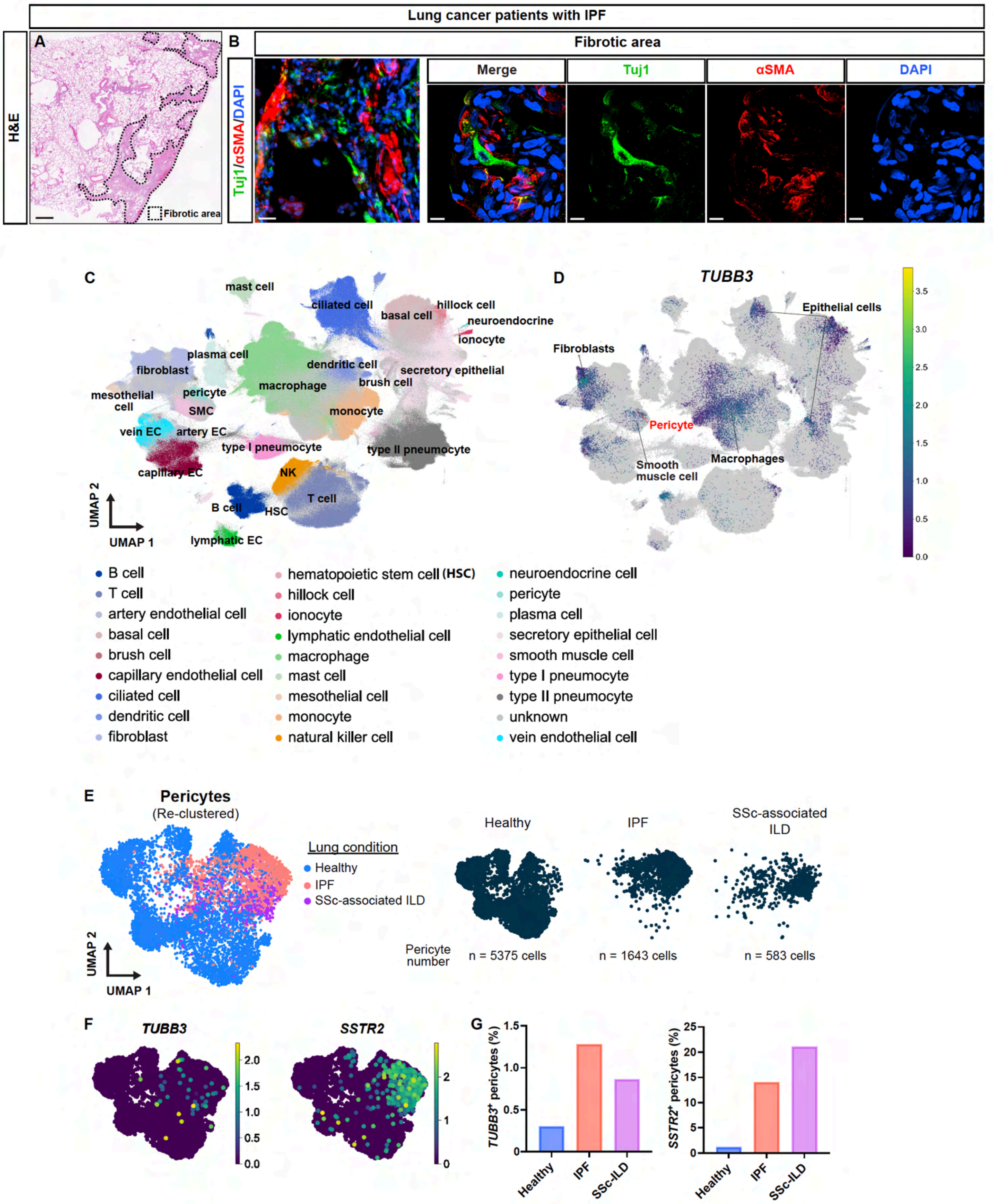

Lung cancer patients with IPF
H&E
A
Fibrotic area
B
Fibrotic area
Tuj1/αSMA/DAPI
Merge
Tuj1
αSMA
DAPI
C
mast cell
ciliated cell
basal cell
hillock cell
neuroendocrine
ionocyte
plasma cell
fibroblast
dendritic cell
secretory epithelial
brush cell
macrophage
mesothelial cell
pericyte
SMC
monocyte
vein EC
artery EC
type I pneumocyte
type II pneumocyte
capillary EC
NK
T cell
B cell
HSC
lymphatic EC
UMAP 2
UMAP 1
D
TUBB3
Epithelial cells
Fibroblasts
Pericyte
Macrophages
Smooth muscle cell
3.5
3.0
2.5
2.0
1.5
1.0
0.5
0.0
B cell
T cell
artery endothelial cell
basal cell
brush cell
capillary endothelial cell
ciliated cell
dendritic cell
fibroblast
hematopoietic stem cell (HSC)
hillock cell
ionocyte
lymphatic endothelial cell
macrophage
mast cell
mesothelial cell
monocyte
natural killer cell
neuroendocrine cell
pericyte
plasma cell
secretory epithelial cell
smooth muscle cell
type I pneumocyte
type II pneumocyte
unknown
vein endothelial cell
E
Pericytes
(Re-clustered)
Lung condition
Healthy
IPF
SSc-associated ILD
UMAP 2
UMAP 1
Healthy
IPF
SSc-associated ILD
Pericyte number
n = 5375 cells
n = 1643 cells
n = 583 cells
F
TUBB3
SSTR2
2.0
1.5
1.0
0.5
0.0
2
1
0
G
TUBB3+ pericytes (%)
1.5
1.0
0.5
0
Healthy
IPF
SSc-ILD
SSTR2+ pericytes (%)
25
20
15
10
5
0
Healthy
IPF
SSc-ILD

Figure S4. ***TUBB3* expression in human pulmonary fibrosis patients.**

(A and B) Analysis of fibrotic lung tissues resected from lung cancer patients with idiopathic pulmonary fibrosis (IPF). **(A)** Hematoxylin and eosin (H&E) staining of the fibrotic lung tissues. Scale bar, 1000 μm. **(B)** Immunostaining of the fibrotic lung tissues with Tuj1 (green), αSMA (red), and DAPI (blue). Left panel shows a low-magnification image, while right panels present high-magnification images of a representative fibrotic region. Scale bars, 30 μm (left); 10 μm (right). **(C and D)** scRNA-seq analysis of whole lung cells from the HLCA datasets. **(C)** UMAP plot of all cells from the HLCA dataset with cell type annotations. **(D)** UMAP plot showing *TUBB3* expression in pericytes, smooth muscle cells, fibroblasts, epithelial cells, and macrophages. **(E–G)** scRNA-seq analysis of pericyte population from the HLCA datasets. **(E)** UMAP plots showing pericyte population from all lung conditions combined (left) and three individual lung conditions shown in separate plots (right). SSc-associated ILD: systemic sclerosis-associated interstitial lung disease. **(F)** UMAP plots showing expression of *TUBB3* and *SSTR2* in pericyte population in all lung conditions. **(G)** Proportion of *TUBB3*$^+$ pericytes (left) and *SSTR2*$^+$ pericytes (right) among total pericytes in each of the three lung conditions.

Figure S5.

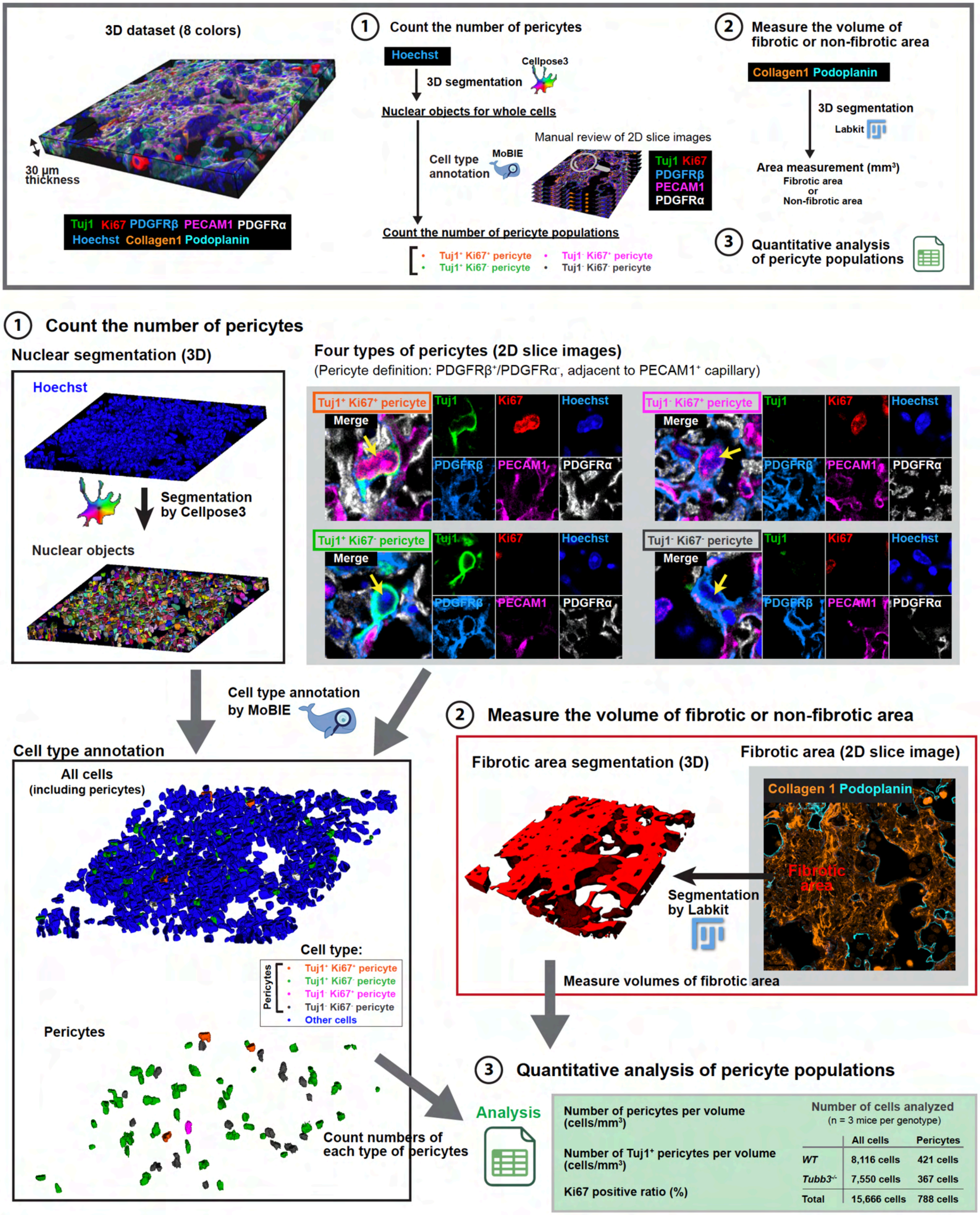
3D dataset (8 colors)
30 µm thickness
Tuj1 Ki67 PDGFRβ PECAM1 PDGFRα
Hoechst Collagen1 Podoplanin
1 Count the number of pericytes
Hoechst
3D segmentation
Cellpose3
Nuclear objects for whole cells
Cell type annotation
MoBIE
Manual review of 2D slice images
Tuj1 Ki67
PDGFRβ
PECAM1
PDGFRα
Count the number of pericyte populations
Tuj1⁺ Ki67⁺ pericyte
Tuj1⁺ Ki67⁻ pericyte
Tuj1⁻ Ki67⁺ pericyte
Tuj1⁻ Ki67⁻ pericyte
2 Measure the volume of fibrotic or non-fibrotic area
Collagen1 Podoplanin
3D segmentation
Labkit
Area measurement (mm³)
Fibrotic area
or
Non-fibrotic area
3 Quantitative analysis of pericyte populations
1 Count the number of pericytes
Nuclear segmentation (3D)
Hoechst
Segmentation by Cellpose3
Nuclear objects
Four types of pericytes (2D slice images)
(Pericyte definition: PDGFRβ⁺/PDGFRα⁻, adjacent to PECAM1⁺ capillary)
Tuj1⁺ Ki67⁺ pericyte
Tuj1⁻ Ki67⁺ pericyte
Tuj1⁺ Ki67⁻ pericyte
Tuj1⁻ Ki67⁻ pericyte
Merge
Tuj1
Ki67
Hoechst
PDGFRβ
PECAM1
PDGFRα
Cell type annotation by MoBIE
Cell type annotation
All cells
(including pericytes)
Cell type:
Pericytes
Tuj1⁺ Ki67⁺ pericyte
Tuj1⁺ Ki67⁻ pericyte
Tuj1⁻ Ki67⁺ pericyte
Tuj1⁻ Ki67⁻ pericyte
Other cells
Pericytes
Count numbers of each type of pericytes
2 Measure the volume of fibrotic or non-fibrotic area
Fibrotic area segmentation (3D)
Fibrotic area (2D slice image)
Collagen 1 Podoplanin
Fibrotic area
Segmentation by Labkit
Measure volumes of fibrotic area
3 Quantitative analysis of pericyte populations
Analysis
Number of pericytes per volume (cells/mm³)
Number of Tuj1⁺ pericytes per volume (cells/mm³)
Ki67 positive ratio (%)
Number of cells analyzed
(n = 3 mice per genotype)
All cells
Pericytes
WT 8,116 cells 421 cells
Tubb3⁻/⁻ 7,550 cells 367 cells
Total 15,666 cells 788 cells

Figure S5. **Workflow for quantitative analysis of pericytes from 3D image dataset.**
Tissue sections stained for eight markers (Tuj1, Ki67, PDGFRβ, PECAM1, PDGFRα, Hoechst, collagen 1, and podoplanin) were imaged in 3D at approximately 30 μm thickness and analyzed using a three-step workflow (summarized at the top): (1) Count the number of pericytes, (2) Measure the volume of fibrotic or non-fibrotic area, and (3) Quantitative analysis of pericyte populations. (1) Count the number of pericytes: All cell nuclei were segmented using deep learning with Cellpose3 from the Hoechst channel, and each segmented object was assigned an ID. This labeled image and the original 8-channel image were imported into MoBIE, where all cells received cell type annotations (classified into five types: $Tuj1^{+}/Ki67^{+}$, $Tuj1^{+}/Ki67^{-}$, $Tuj1^{-}/Ki67^{+}$, $Tuj1^{-}/Ki67^{-}$ pericytes, and other cells). Pericytes were defined as $PDGFR\beta^{+}/PDGFR\alpha^{-}$ cells attached to $PECAM1^{+}$ capillaries, and their Tuj1 and Ki67 expression status was assessed during annotation. (2) Measure the volume of fibrotic or non-fibrotic area: Fibrotic and non-fibrotic areas were 3D segmented using machine learning in Fiji's labkit based on collagen1 and podoplanin channels. The volume of each area was calculated from the segmented images. (3) Quantitative analysis of pericyte populations: Data from steps 1 and 2 were used to calculate the number of pericytes per volume, number of $Tuj1^{+}$ pericytes per volume, and $Ki67^{+}$ ratio in pericytes. This analysis evaluated a total of 15,666 cells from three *WT* and three *Tubb3*$^{-/-}$ mice each.

Figure S6.

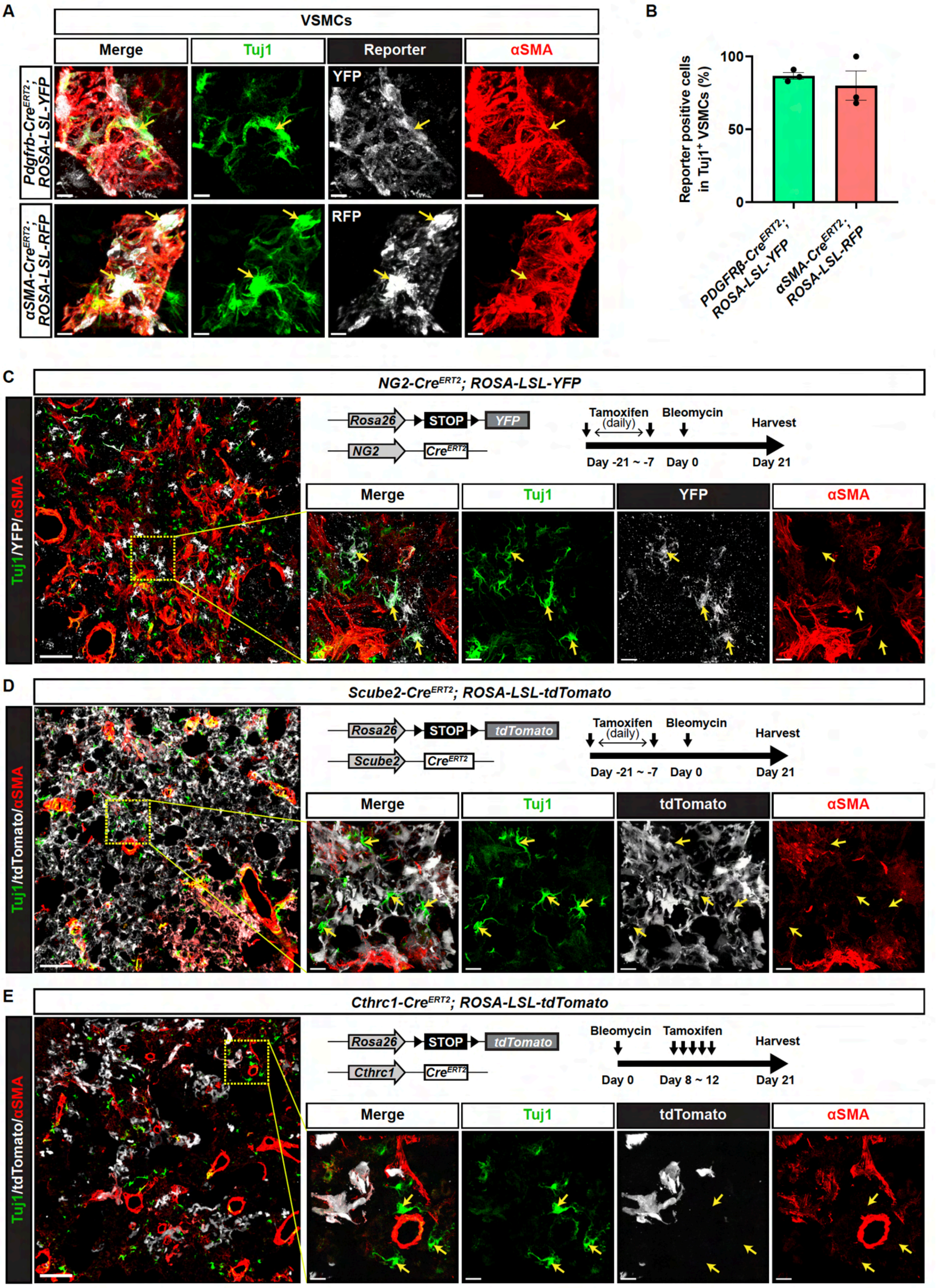
A
VSMCs
Merge
Tuj1
Reporter
αSMA
Pdgfrb-CreERT2; ROSA-LSL-YFP
YFP
αSMA-CreERT2; ROSA-LSL-RFP
RFP
B
Reporter positive cells in Tuj1+ VSMCs (%)
100
50
0
PDGFRβ-CreERT2; ROSA-LSL-YFP
αSMA-CreERT2; ROSA-LSL-RFP
C
NG2-CreERT2; ROSA-LSL-YFP
Tuj1/YFP/αSMA
Rosa26
STOP
YFP
NG2
CreERT2
Tamoxifen (daily)
Bleomycin
Harvest
Day -21 ~ -7
Day 0
Day 21
Merge
Tuj1
YFP
αSMA
D
Scube2-CreERT2; ROSA-LSL-tdTomato
Tuj1/tdTomato/αSMA
Rosa26
STOP
tdTomato
Scube2
CreERT2
Tamoxifen (daily)
Bleomycin
Harvest
Day -21 ~ -7
Day 0
Day 21
Merge
Tuj1
tdTomato
αSMA
E
Cthrc1-CreERT2; ROSA-LSL-tdTomato
Tuj1/tdTomato/αSMA
Rosa26
STOP
tdTomato
Cthrc1
CreERT2
Bleomycin
Tamoxifen
Harvest
Day 0
Day 8 ~ 12
Day 21
Merge
Tuj1
tdTomato
αSMA

Figure S6. **Lineage-tracing experiments revealed that neither VSMCs nor fibroblasts contribute to Tuj1$^+$ pericytes.**

(A and B) Lineage-tracing experiments of VSMCs using *Pdgfrb-Cre^{ERT2}; ROSA-LSL-YFP* and *αSMA-Cre^{ERT2}; ROSA-LSL-RFP* mice. **(A)** Immunostaining of bleomycin-treated lungs from *Pdgfrb-Cre^{ERT2}; ROSA-LSL-YFP* (upper panels) and *αSMA-Cre^{ERT2}; ROSA-LSL-RFP* (lower panels) mice, with Tuj1 (green), lineage-specific reporter protein (gray), and αSMA (red). Scale bars, 5 μm. **(B)** Proportion of YFP- or RFP-expressing cells within Tuj1$^+$ VSMCs. **(C)** Lineage-tracing experiments using *NG2-Cre^{ERT2}; ROSA-LSL-YFP* mice. The Cre-mediated excision was induced by administrating tamoxifen for 14 days prior to initiating bleomycin treatment, with lung tissues harvested on day 21. Immunostaining of bleomycin-treated lungs with Tuj1 (green), YFP (gray), and αSMA (red). Boxed region in the left panel is magnified in the right panels. Arrows indicate Tuj1$^+$ pericytes. Scale bars, 100 μm (left); 15 μm (magnified views). **(D)** Lineage-tracing experiments using *Scube2-Cre^{ERT2}; ROSA-LSL-tdTomato* mice. The Cre-mediated excision was induced by administrating tamoxifen for 14 days prior to initiating bleomycin treatment, with lung tissues harvested on day 21. Immunostaining of bleomycin-treated lungs with Tuj1 (green), tdTomato (gray), and αSMA (red). Boxed region in the left panel is magnified in the right panels. Arrows indicate Tuj1$^+$ pericytes. Scale bars, 100 μm (left); 15 μm (magnified views). **(E)** Lineage-tracing experiments using *Cthrc1-Cre^{ERT2}; ROSA-LSL-tdTomato* mice. The Cre-mediated excision was induced by administrating tamoxifen for 5 days, starting 8 days post-bleomycin treatment, with lung tissues harvested on day 21. Immunostaining of bleomycin-treated lungs with Tuj1 (green), tdTomato (gray), and αSMA (red). Boxed region in the left panel is magnified in the right panels. Arrows indicate Tuj1$^+$ pericytes. Scale bars, 100 μm (left); 15 μm (magnified views).

Figure S7.

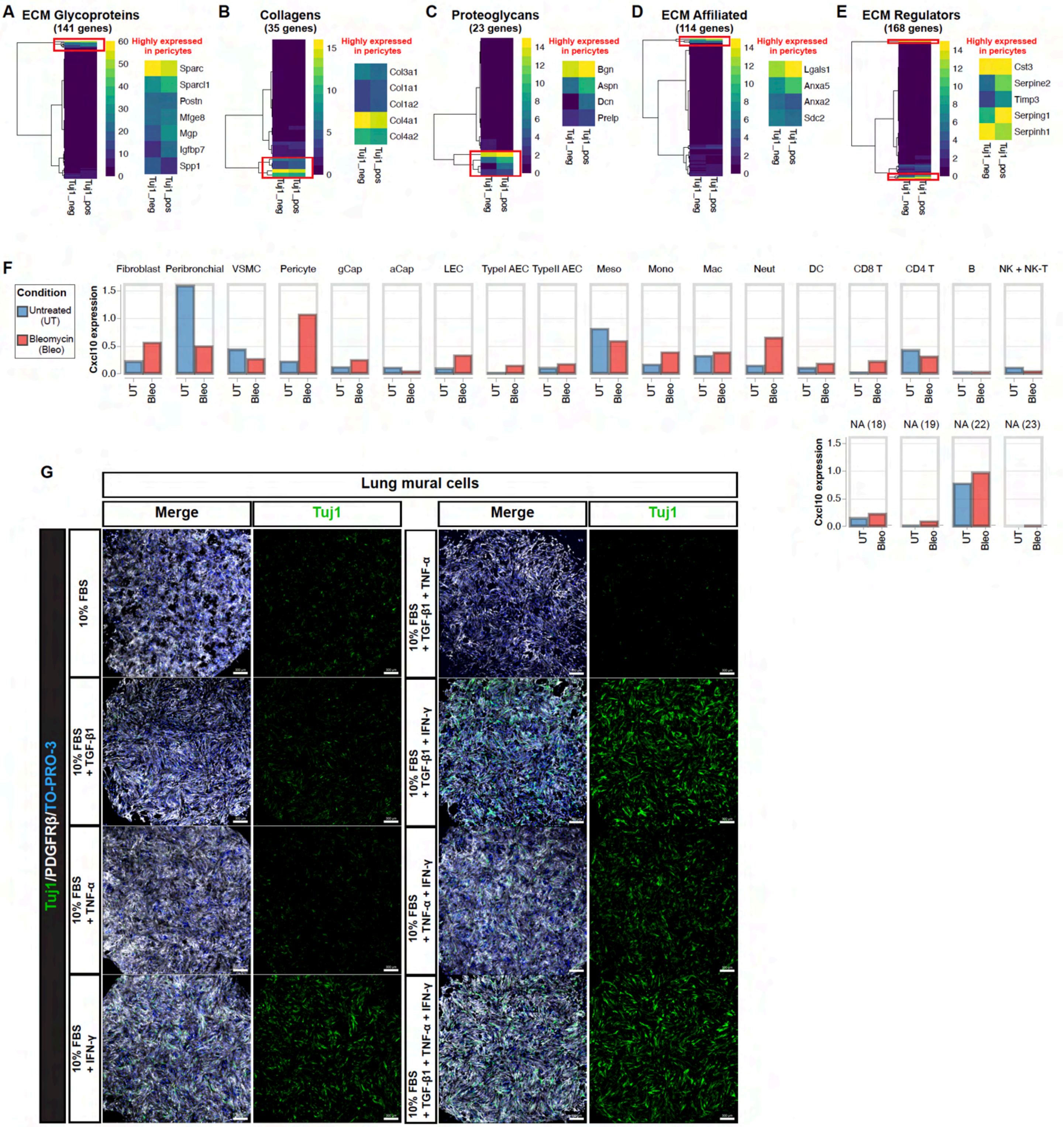

Figure S7. **Gene expression profiles and upstream signals regulating Tuj1 in lung pericytes.** **(A–E)** Heatmap of the average expression levels for matrisome genes in Tuj1^{neg} and Tuj1^{pos} pericytes from bleomycin-treated lung samples, based on scRNA-seq data[25]. Matrisome categories displayed: (A) ECM Glycoproteins, (B) Collagens, (C) Proteoglycans, (D) ECM Affiliated, and (E) ECM Regulators. For each category, a highly expressed gene cluster (highlighted by a red rectangle) is enlarged on the right, with corresponding gene names shown. ECM: extracellular matrix. **(F)** Average expression of *Cxcl10* in various cell types from untreated (blue) and bleomycin-treated (red) samples of the scRNA-

seq dataset from *Tsukui et al.,* 2020[25]. Cell type annotations are based on the original publication. UT: untreated; Bleo: bleomycin-treated; Peribronchial: peribronchial fibroblast; VSMC: vascular smooth muscle cell; gCap: general capillary cell; aCap: aerocyte; LEC: lymphatic endothelial cell; TypeI AEC: type I alveolar epithelial cell; TypeII AEC: type 2 alveolar epithelial cell; Meso: mesothelial cell; Mono: monocyte; Mac: macrophage; Neut: neutrophil; DC: dendritic cell; CD8 T: CD8 T cell; CD4 T: CD4 T cell; B: B cell; NK + NK-T: natural killer cell + natural killer T cell; NA: not applicable. **(G)** Immunostaining of cultured lung mural cells with PDGFRβ (gray), Tuj1 (green), and TO-PRO-3 (blue). The freshly isolated lung mural cells were cultured for 48 hours in growth medium containing 10% fetal bovine serum (FBS), either alone or supplemented with IFN-γ, TGF-β1 or TNF-α, pairwise combinations of these cytokines, or all these cytokines together. Note that the combination of IFN-γ and TGF-β1 effectively induced Tuj1 expression in lung mural cells in the primary culture. Scale bars, 300 μm.

Figure S8.

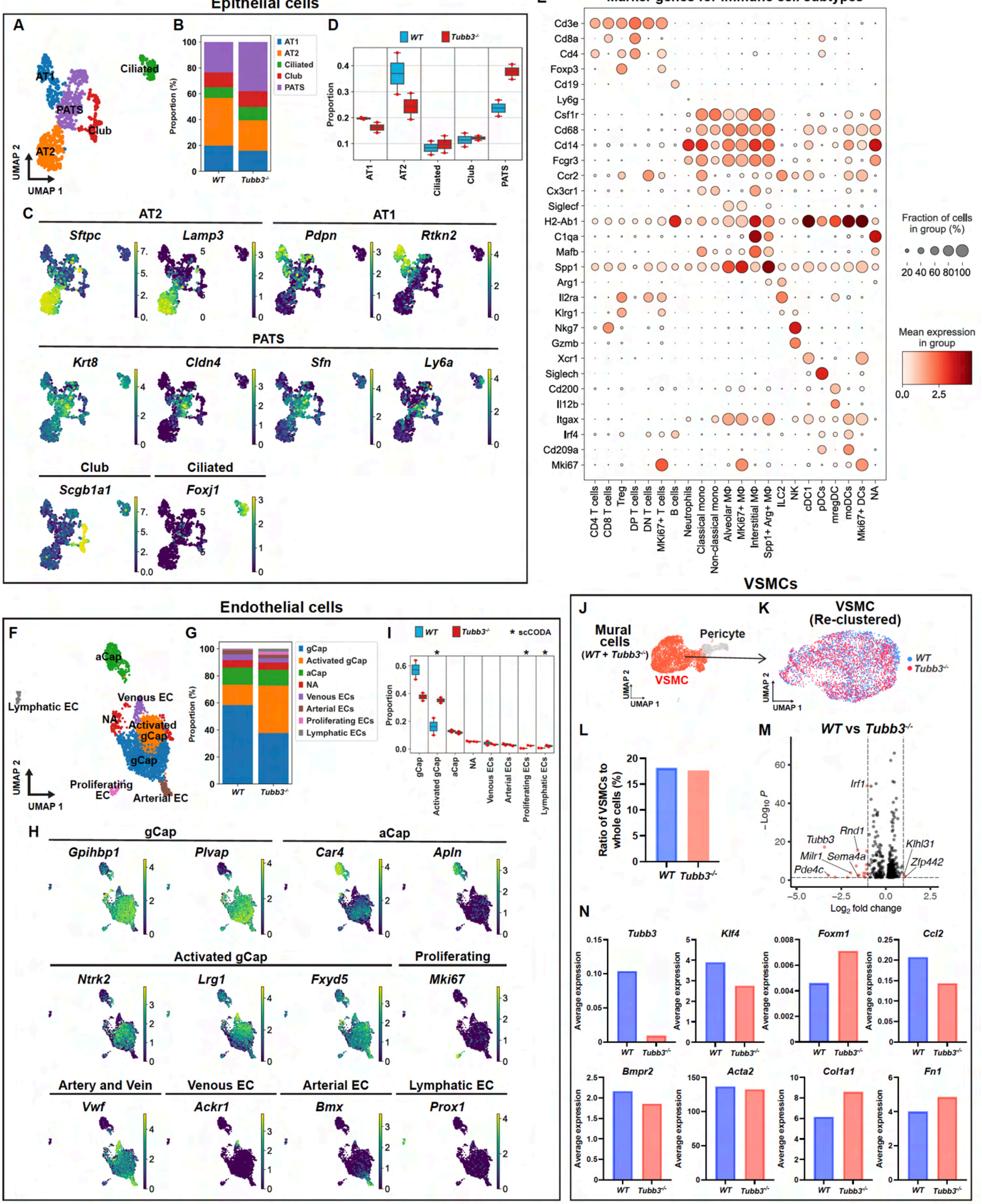
Epithelial cells
A
AT1
Ciliated
PATS
Club
AT2
UMAP 2
UMAP 1
B
Proportion (%)
AT1
AT2
Ciliated
Club
PATS
WT
Tubb3-/-
D
WT
Tubb3-/-
Proportion
AT1
AT2
Ciliated
Club
PATS
C
AT2
AT1
Sftpc
Lamp3
Pdpn
Rtkn2
PATS
Krt8
Cldn4
Sfn
Ly6a
Club
Ciliated
Scgb1a1
Foxj1
E
Marker genes for immune cell subtypes
Cd3e
Cd8a
Cd4
Foxp3
Cd19
Ly6g
Csf1r
Cd68
Cd14
Fcgr3
Ccr2
Cx3cr1
Siglecf
H2-Ab1
C1qa
Mafb
Spp1
Arg1
Il2ra
Klrg1
Nkg7
Gzmb
Xcr1
Siglech
Cd200
Il12b
Itgax
Irf4
Cd209a
Mki67
CD4 T cells
CD8 T cells
Treg
DP T cells
DN T cells
Mki67+ T cells
B cells
Neutrophils
Classical mono
Non-classical mono
Alveolar MΦ
Mki67+ MΦ
Interstitial MΦ
Spp1+ Arg+ MΦ
ILC2
NK
cDC1
pDCs
mregDC
moDCs
Mki67+ DCs
NA
Fraction of cells in group (%)
20 40 60 80100
Mean expression in group
0.0
2.5
Endothelial cells
F
aCap
Lymphatic EC
Venous EC
NA
Activated gCap
gCap
Proliferating EC
Arterial EC
UMAP 2
UMAP 1
G
Proportion (%)
gCap
Activated gCap
aCap
NA
Venous ECs
Arterial ECs
Proliferating ECs
Lymphatic ECs
WT
Tubb3-/-
I
WT
Tubb3-/-
* scCODA
Proportion
gCap
Activated gCap
aCap
NA
Venous ECs
Arterial ECs
Proliferating ECs
Lymphatic ECs
H
gCap
aCap
Gpihbp1
Plvap
Car4
Apln
Activated gCap
Proliferating
Ntrk2
Lrg1
Fxyd5
Mki67
Artery and Vein
Venous EC
Arterial EC
Lymphatic EC
Vwf
Ackr1
Bmx
Prox1
VSMCs
J
Mural cells
(WT + Tubb3-/-)
Pericyte
VSMC
UMAP 2
UMAP 1
K
VSMC
(Re-clustered)
WT
Tubb3-/-
UMAP 2
UMAP 1
L
Ratio of VSMCs to whole cells (%)
WT
Tubb3-/-
M
WT vs Tubb3-/-
−Log10 P
Irf1
Rnd1
Tubb3
Klhl31
Milr1
Sema4a
Zfp442
Pde4c
Log2 fold change
N
Tubb3
Klf4
Foxm1
Ccl2
Average expression
WT
Tubb3-/-
Bmpr2
Acta2
Col1a1
Fn1

Figure S8. **scRNA-seq analysis of epithelial cells, endothelial cells, and VSMCs in *WT* and *Tubb3$^{-/-}$* mice.**

(A–D) Cell compositional analysis of epithelial populations from scRNA-seq data of *WT* and *Tubb3$^{-/-}$* mice 14 days after bleomycin treatment. UMAP plot of epithelial cells showing 5 subtypes (A) as defined by marker genes (C). **(B and D)** Proportions of each subtype relative to the total epithelial cells in *WT* and *Tubb3$^{-/-}$* mice are shown as stacked barplot (B) and boxplot (D). No significant changes were observed by scCODA analysis in panel (D). AT1: alveolar type I cell; AT2: alveolar type II cell; PATS: pre-alveolar type-1 transitional cell state cell; Club: club cell; Ciliated: ciliated cell. **(E)** Dot plot showing expression of marker genes for immune cell subtypes from scRNA-seq data of bleomycin-treated lungs (day 14) from *WT* and *Tubb3$^{-/-}$* mice. **(F–I)** Cell compositional analysis of endothelial populations from scRNA-seq data of *WT* and *Tubb3$^{-/-}$* mice 14 days after bleomycin treatment. UMAP plot of endothelial cells showing 7 subtypes (F) as defined by marker genes (H). **(G and I)** Proportions of each subtype relative to the total endothelial cells in *WT* and *Tubb3$^{-/-}$* mice are shown as stacked barplot (G) and boxplot (I). Statistically credible changes, as tested by scCODA, are noted with an * in panel (I). gCap: general capillary cell; aCap: aerocyte; EC: endothelial cell; NA: not applicable. **(J)** UMAP plot of mural cell subtypes (VSMC and pericyte) from the scRNA-seq analysis of *WT* and *Tubb3$^{-/-}$* mice. **(K-N)** scRNA-seq analysis of the VSMC population. **(K)** UMAP plot of re-clustered *WT* VSMC (blue) and *Tubb3$^{-/-}$* VSMC (red) population. **(L)** Percentage of *WT* and *Tubb3$^{-/-}$* VSMCs relative to total lung cells. **(M)** Volcano plot showing differentially expressed genes (DEGs) between *WT* (left) and *Tubb3$^{-/-}$* (right) VSMCs. Red dots represent genes with $\log_2$FC > 1 and adjusted $P < 0.05$. **(N)** Average expression levels of *Tubb3*, *Klf4*, *Foxm1*, *Ccl2*, *Bmpr2*, *Acta2*, *Col1a1*, and *Fn1* in *WT* and *Tubb3$^{-/-}$* pericytes.

Table S1. Lists of primary and secondary antibodies for immunostaining

Primary antibody	Company	Catalog number	Host	Dilution	RRID
Tuj1-Alexa 488	Biolegend	801203	Mouse	1:200	AB_2564757
Tuj1-Alexa 647	Biolegend	801209	Mouse	1:200	AB_2686930
aSMA-FITC	Millipore Sigma	F3777	Mouse	1:500	AB_476977
aSMA-Cy3	Millipore Sigma	C6198	Mouse	1:500	AB_476856
PECAM1	Chemicon	MAB1398z	hamster	1:300	AB_94207
EpCAM	Biolegend	118201	Rat	1:100	AB_1089026
PDGFRb	eBioscience	14-1402-82	Rat	1:100	AB_467493
PDGFRβ	R&D	AF1042-SP	Goat	1:100	AB_2162633
NG2	Millipore Sigma	AB5320	Rabbit	1:200	AB_91789
MCAM-Alexa 647	Biolegend	134717	Rat	1:100	AB_2721426
GFP	Thermo Fisher	A-11122	Rabbit	1:1000	AB_221569
GFP	Abcam	ab6673	Goat	1:500	AB_305643
DsRed	TaKaRa	632496	Rabbit	1:1000	AB_10013483
Collagen 1	Millipore Sigma	AB765P	Rabbit	1:200	AB_92259
Podoplanin-Alexa 594	Biolegend	127414	Syrian hamster	1:100	AB_2563351
Podoplanin-APC	Biolegend	127410	Syrian hamster	1:100	AB_10613649
TrkB	R&D	AF1494-SP	Goat	1:200	AB_2155264
PDGFRa	R&D	AF1062-SP	Goat	1:200	AB_2236897
CD45	eBioscience	14-0451-85	Rat	1:500	AB_467252
CD68-Alexa 488	Biolegend	137011	Rat	1:100	AB_2074847
CD68-Alexa 594	Biolegend	137020	Rat	1:100	AB_2563305
Arginase-PE	Thermo Fisher	12-3697-80	Rat	1:200	AB_2734838
SPP1	R&D	AF808-SP	Goat	1:400	AB_2194992
Ki67-Alexa 488	BD	558616	Mouse	1:100	AB_647087
PGP9.5	Abcam	ab15503	Rabbit	1:200	AB_301912

TH	Millipore Sigma	AB152	Rabbit	1:200	AB_390204
CSF1R	Santa Cruz	sc-692	Rabbit	1:400	AB_631025

Secondary antibody	Company	Catalog number	Host	Dilution	RRID
anti Armenian hamster-Alexa 488	Jackson ImmunoResearch	127-545-160	Goat	1:250	AB_2338997
anti Armenian hamster-Cy3	Jackson ImmunoResearch	127-165-160	Goat	1:250	AB_2338989
anti Armenian hamster-Alexa 647	Jackson ImmunoResearch	127-605-160	Goat	1:250	AB_2339001
anti Goat-Alexa 488	Jackson ImmunoResearch	705-547-003	Donkey	1:250	AB_2340431
anti Goat-Cy3	Jackson ImmunoResearch	705-167-003	Donkey	1:250	AB_2340414
anti Goat-Alexa 594	Jackson ImmunoResearch	705-585-147	Donkey	1:250	AB_2340433
anti Rabbit-Alexa 488	Invitrogen	A11034	Goat	1:250	AB_2576217
anti Rabbit-Cy3	Jackson ImmunoResearch	711-165-152	Donkey	1:250	AB_2307443
anti Rabbit-Alexa 647	NanoTag	N2404	Alpaca	1:250	AB_3076043
anti Rat-Cy3	Jackson ImmunoResearch	712-166-153	Donkey	1:250	AB_2340669
